# Pilot Study of Diagnostic Performances of Vascular Biomarkers Soluble fms-Like Tyrosine Kinase and Placental Growth Factor in Scleroderma Renal Crisis

**DOI:** 10.1016/j.ekir.2024.12.025

**Published:** 2024-12-31

**Authors:** Aïcha Kante, Paul Legendre, Bérangère S. Joly, Bertrand Dunogué, Alexandre Hertig, Benjamin Terrier, Elodie Massolin, Paul Coppo, Felix Ackermann, Giorgina Barbara Piccoli, Luc Mouthon, Jean Guibourdenche, Benjamin Chaigne

**Affiliations:** 1 Service de Médecine Interne, Centre de Référence Maladies Systémiques Autoimmunes et Autoinflammatoires Rares d'Ile de France de l’Est et de l’Ouest, Hôpital Cochin, Assistance Publique-Hôpitaux de Paris, Paris, France; 2 APHP-CUP, Hôpital Cochin, Université Paris Cité, Paris, France; 3 Sorbonne Université, Paris, France; 4 Service d’Immunologie clinique, Centre hospitalier du Mans, Le Mans, France; 5 Service d’Hématologie Biologique, Hôpital Lariboisière, Assistance Publique-Hôpitaux de Paris (APHP Nord), Université Paris Cité, Paris, France; 6 INSERM UMRS-1138, Équipe 16, Centre de Recherche des Cordeliers, Université Paris Cité, Paris, France; 7 Centre National de Référence des Microangiopathies Thrombotiques, Paris, France; 8 Service de Néphrologie, Hôpital Foch, Suresnes, France; 9 UF d'Hormonologie, Hôpital Cochin, Assistance Publique-Hôpitaux de Paris (APHP), Paris, France; 10 Service d'Hématologie, Hôpital Saint-Antoine, AP-HP, Paris, France; 11 Service de Médecine interne, Hôpital Foch, Suresnes, France

**Keywords:** endotheliosis, microangiopathy, PlGF, scleroderma renal crisis, sFlt-1, systemic sclerosis

## Abstract

**Introduction:**

Scleroderma renal crisis (SRC) is a major vascular complication of systemic sclerosis (SSc), associated with high morbidity and mortality. In this retrospective study, we evaluated the potential prognostic and diagnostic roles of angiogenesis molecules, placental growth factor (PlGF), soluble fms-like tyrosine kinase 1 (sFlt-1) and sFlt1/PlGF ratio as biomarkers in SRC.

**Methods:**

Sera samples from 27 patients with a history of SRC (SSc-SRC+) were collected following event occurence. Biomarker levels were assessed using an electrochemiluminescence immunoassay and compared with age- and sex-matched patients with SSc-SRC− (*n* = 24), hemolytic uremic syndrome (HUS) (*n* = 27), malignant hypertension (MHT) (*n* = 22), and donors (*n* = 61). Areas under the receiver-operating-characteristic curves (AUC) were used to evaluate diagnostic accuracy. Long-term dialysis risk was evaluated using a Cox model.

**Results:**

The median (interquartile range [IQR]) PlGF (pg/ml) was significantly higher in the serum of patients with SSc-SRC+ (42.1 [21.4–51.8]) compared with donors (14.7 [11.8–17.9]), those with SSc-SRC− (18.5 [14.7–21.5]) (*P* < 0.0001), those with HUS (22.8 [19.5–29.6]), and those with MHT (25.5 [17.2–39.3]) (*P* < 0.0001). In a multivariate regression adjusting for multiple confounders, PlGF was associated with higher SRC risk with an odds ratio of 1.08 [1.01–1.22], (*P* = 0.034). A PlGF level above 24.5 pg/ml revealed an AUC of 0.81 (confidence interval [0.68–0.94]), a specificity of 95%, and a sensitivity of 67% for SRC diagnosis. Eleven patients with SSc-SRC+ reached end-stage kidney failure with significantly higher PlGF (42.9 [22.4–78.2]) compared with patients who were dialysis-free (19.7 [15.6–29.7], *P* = 0.03).

**Conclusion:**

Serum PlGF may identify the risk of SRC occurrence among patients with SSc with a good specificity and represents a potential tool for long-term dialysis risk evaluation.

## Graphical abstract

**Figure:**
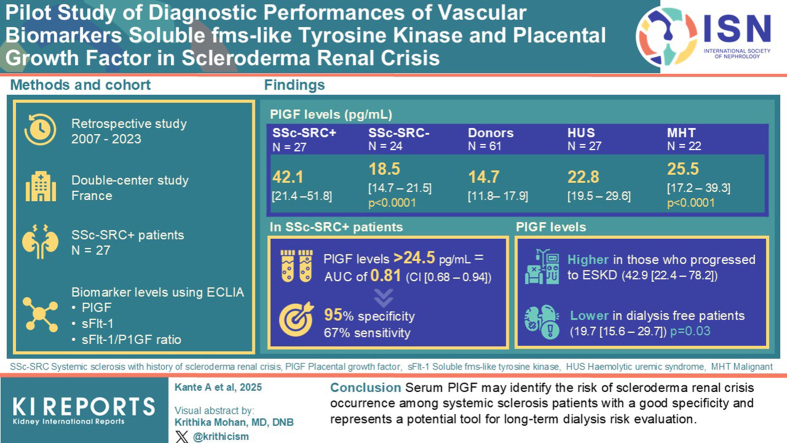

Scleroderma renal crisis (SRC) is a major life-threatening complication of systemic sclerosis (SSc).^1^^,^^2^ SRC is defined by the new onset of hypertension with a blood pressure value > 140/90 mmHg or a > 30 mmHg increase of systolic blood pressure from baseline, acute kidney injury (increase in serum creatinine to 1.5 times baseline or more, or by at least 0.3 mg/dl [26.5 μmol/l]) according to the Kidney Disease Improving Global Outcomes criteria, and microangiopathic hemolytic anemia, associated with other organ dysfunction in the context of SSc.^3^ Over the past decades, several predictive factors for SRC have been identified such as diffuse cutaneous SSc (dcSSc), rapid progression of skin thickening, disease onset within the first 4 years, new cardiac events or anemia, and prednisone treatment > 15 mg/d within the last 3 months. Among them, the presence of anti-RNA polymerase III is the most robust risk factor for SRC.^4^^,^^5^ Before the introduction of angiotensin-converting enzyme inhibitors in 1980, SRC was the primary cause of mortality among patients with SSc.^6^ Although survival has significantly improved with angiotensin-converting enzyme inhibitor treatment and global awareness against the use of high-dose corticosteroids, SRC remains a life-threatening condition with a 20% 6-month mortality rate^7^ and poses a 19% to 42% risk of developing chronic kidney disease requiring dialysis.^3^^,^^8^ During SRC, susceptible individuals develop an endothelial injury and vascular remodeling with narrowing of arterial lumens, provoking glomerular ischemia which in turn leads to the activation of the renin-aldosterone axis^9^ and provokes an imbalance of proangiogenic and antiangiogenic factors.^2^ Although many attempts have been made to identify a biomarker that could predict SRC, none of them except for the presence of anti-RNA polymerase III antibodies^5^ can be used in clinical practice, and no biomarker has been previously linked to renal prognosis following SRC.

Factors regulating angiogenesis, including sFlt-1, PlGF, and their ratio, have been extensively studied in preeclampsia, a potentially life-threatening disease that also involves endothelial injury.^10^ In SSc, sFlt-1, and PlGF have been previously identified as potential biomarkers of pulmonary hypertension (PH) in a prospective cohort study of 300 patients, with higher levels of PlGF and sFlt-1 in patients with PH compared with patients with SSc without PH, respectively.^11^

To our knowledge, the links between sFlt-1, PlGF, and SRC occurrence, have not been studied. Herein, we aimed to study the diagnostic performance and prognostic values of vascular biomarkers, sFlt-1, PlGF, and their ratio in SRC.

## Methods

### Study Design

We conducted a retrospective study of patients with SSc who were followed up in the Department of Internal Medicine at Cochin University Hospital (Assistance Publique – Hôpitaux de Paris, Paris France, a national referral center for rare systemic autoimmune and autoinflammatory disease in Ile de France, East and West). This study was conducted according to the Strengthening the Reporting of Observational Studies in Epidemiology reporting guidelines for cohort studies.

### Subjects – Patients

#### SRC Patients

Sera samples contained in the Cochin University Hospital Biobank were obtained from patients fulfilling the 2013 American College of Rheumatology and the European Alliance of Associations for Rheumatology classification criteria for SSc.^12^ Samples were collected between 2007 and 2023 at the time closest to the onset of SRC, after the occurrence of the event. PlGF measurements were not performed before the onset of SRC. Cases (SSc-SRC+) were defined as patients with SSc who presented with SRC according to the classification criteria defined by Butler *et al.*, which include a new onset hypertension, a documented recent increase in serum creatinine levels in the absence of a differential diagnosis for acute kidney injury, as well as microangiopathic hemolytic anemia and related target organ dysfunctions such as hypertensive retinopathy, hypertensive encephalopathy, acute heart failure, or acute pericarditis.^13^ Patients with available samples and clinical data, fulfilling criteria for SRC were included in the study.

#### Control Patients

Each SSc-SRC+ (*n* = 27) patient was compared with age-matched (categories ± 5 years) and sex-matched SSc-SRC− controls (*n* = 24), hemolytic uremic syndrome (HUS, *n* = 27), patients with malignant hypertension (MHT, *n* = 22), and donors (*n* = 61) assessed through database searching (Cochin University Hospital for patients with SSc and donors, Lariboisière University Hospital for HUS and MHT). “Sex” referred to phenotypical characteristics and was extracted from electronic health records. Patients with limited cutaneous SSc were defined by skin thickening in areas beyond the elbows and the knees, whereas patients with dcSSc were defined by skin thickening proximal, as well as distal to the elbows and the knees.^14^ HUS was defined as the combination of hemolytic anemia, acute kidney injury, and low platelet count, with the additional criterion of preserved (> 20%) activity of ADAMTS13, thereby ruling out thrombotic thrombocytopenic purpura. MHT was defined by elevated blood pressure > 180/110 mmHg with concomitant diffuse microvascular injury (acute kidney injury, microangiopathic hemolytic anemia, or high-grade retinopathy) according to the European consensus.^15^ Clinical data were collected at the time closest to blood sample collection and included arterial blood pressure, kidney function, and hemolysis biomarkers. Donors’ sera were collected from the Hormonology Functional Unit biobank (Cochin University Hospital), and included individuals without any cardiovascular, autoimmune, or inflammatory conditions.

### Data Collection

Baseline was defined as the date of inclusion in the Cochin University Hospital scleroderma database, and follow-up as the time between serum sample acquisition and the last available visit at the time of data extraction. Disease duration was calculated from the time of onset of the first SSc-related clinical event (other than Raynaud’s phenomenon). Medical history, physical examinations, laboratory tests including antibody status, and radiology examinations were collected for each patient at the time of the event (for SSc-SRC+, MHC, and HUS patients). Each patient with SSc underwent annual follow-up visits following inclusion, except when complications occurred with ensuing hospitalization. The most recent clinical data available was used to assess the outcomes.

#### Samples

Blood samples from patients with SSc were centrifuged, and serum aliquots were stored at −80 °C until analysis (APHP DMU Biophygen, Centre de Ressources Biologiques Cochin Hospital). Blood samples from control subjects diagnosed with HUS and MHT were retrieved from the biobank of the National Reference Center for Microangiopathic Thrombopathy, housed at the Lariboisière Hospital (biobank registration number: AC-2023-6021).

#### Serum Assay

Samples were thawed and centrifuged immediately before use. sFlt-1 and PlGF levels were measured using electrochemiluminescence immunoassay performed with a COBAS e analyzer (Roche diagnostics, Germany).^16^ This technology is based on a sandwich binding immunoassay using mouse monoclonal antibodies directed against sFlt-1 and PlGF.^17^ Assay ranges were between 10–85,000 pg/ml and 3–10,000 pg/ml, quantification limit between 15 pg/ml and 10 pg/ml, intraassay and interassay variations were < 6% for sFlt-1 and PlGF, respectively. sFlt-1/PlGF ratio was calculated because of its widespread use in the context of preeclampsia.

#### Outcomes

The outcomes were assessed among patients with SSc and included end-stage kidney failure requiring long-term dialysis (which was defined by sustained dialysis for more than 3 months) and kidney transplantation. Outcome data was obtained through the analysis of electronic health records. Potential confounders and effect modifiers for SRC, including anti-RNA polymerase III antibody positivity, steroid treatment and estimated glomerular filtration rates were assessed among all patients with SSc because of their known association with SRC risk.

### Statistical Analyses

Data were reported as numbers (*n*) and percentages (%) for categorial variables and compared using the Chi-square or Fisher exact test as appropriate. Quantitative variables were expressed as median and IQR and compared using the Kruskal–Wallis’ test for multiple group comparison. Mann-Whitney-Wilcoxon sum rank test was used to compare biomarker levels between patients with SSc-SRC+ and those with SSc-SRC−. Statistical analyses were performed using Stata/MP 13.0 (StataCorp, College Station, TX) and Prism 10.0.3 (GraphPad Software, Boston, MA). Receiver-operating-characteristic curves were generated using R statistical software (R Studio Version 2023.12.0 + 369), and optimal thresholds for biomarkers were determined using Youden’s index. A univariate Cox model analysis was performed to assess the risk of long-term dialysis within the SSc population according to biomarker level. Proportional hazard assumption was evaluated using Schoenfeld residuals. The independent associations, adjusted for potential confounders, were evaluated using a penalized multivariate logistic regression analysis with Firth’s correction. Differences were considered significant for a *P*-value < 0.05. Differences were considered significant for a *P*-value < 0.05.

### Ethical Approval

The French SSc national cohort study database received ethical approval from CCTIRS (approval no. 13.145; Advisory Committee on Information Processing in Material Research in the Field of Health). Data protection complied with the requirements of the National Information Science and Liberties Commission (no. 914607). All included patients provided informed consent. Since 2000, all patients presenting with a thrombotic microangiopathy syndrome have been prospectively included in the French Registry (Clinical Department of Professor Paul Coppo, Saint-Antoine Hospital, AP-HP), accredited by the National Rare Diseases Plan of the Ministry of Health since 2006. Each patient provided written consent, in compliance with the Helsinki Declaration, to participate in this study.

## Results

### Patients’ Characteristics

We first extensively assessed the clinical characteristics of patients with SSc. Twenty-seven patients with a history of SRC (SSc-SRC+ group), comprising 17 female and 10 male patients, were included in the study. The clinical and biological characteristics of patients with SSc are summarized in Table 1. As expected, after matching, there were no significant differences in median age (*P* = 0.5) or prevalence of female sex (*P* = 0.8). The median age at disease onset from the first non-Raynaud’s phenomenon symptom was not significantly different (aged 47 years among patients SSc-SRC+ compared with 42 years in the SSc-SRC− group. The median (IQR) disease duration at the time of inclusion was 1 (0–2.5) year among patients with SSc-SRC+. In the SSc-SRC+ group, dcSSc was more prevalent, representing 78% compared with 25% in the SSc-SRC− group (*P* < 0.001). The SSc-SRC+ group had a median modified Rodnan skin score of 27 (11–34) compared with a median of 4 (2–8) (*P* < 0.001) in the group with no history of SRC. Ten patients (37%) in the SSc-SRC+ group had anti-RNA polymerase III antibodies compared with 3 (13%) in the SSc-SRC− group (*P* = 0.04). Twelve patients (50%) in the SSc-SRC− group had anti-centromere antibodies, whereas none of the patients with SSc-SRC+ presented these antibodies (*P* < 0.001). There was no significant difference in treatment with angiotensin-converting enzyme inhibitor before SRC. Eleven patients (47%) had received corticosteroids before SRC episode, compared with 4 (17%) in the SSc-SRC− group (*P* = 0.06). About half of the patients were treated with immunosuppressants in the SSc-SRC+ and in the SSc-SRC− groups. Among the SSc population, patients with SSc-SRC+ had higher median concentrations of serum creatinine at 254 (115–391) μmol/l, compared with the SSc-SRC− group with a median of 66 (59–85) μmol/l (*P* < 0.0001) (Table 2).

**Table 1:** Representation the clinical characteristics between patients with SSc with and without history of SRC

Characteristics	All SSc (*n* = 51)	SSc SRC+ (*n* = 27)	SSc SRC− (*n* = 24)	*P*-value
Population Female, *n (%)*	35 (69)	17 (63)	18 (75)	0.36
Age at diagnosis, median [IQR] (yrs)	45 [34–57]	47 [39–59]	42 [31–47]	0.09
Hypertension, *n (%)*	15 (29)	13 (48)	2 (8)	0.002
Blood pressure (BP)				
Systolic BP, median [IQR] (mmHg)	130 [116–173]	185 [151–210]	120 [110–130]	< 0.001
Diastolic BP, median [IQR] (mmHg)	77 [67–91]	101 [89–119]	70 [66–78]	< 0.001
Clinical presentation				
Diffuse SSc, *n (%)*	27 (53)	21 (78)	6 (25)	< 0.001
mRSS, median [IQR]	10 [2–29]	27 [10–34]	4 [1–9]	< 0.001
Mouth opening, median [IQR], (mm)	35 [28–45]	30 [23–40]	41 [34–48]	< 0.001
Interstitial lung disease, *n (%)*	23 (45)	12 (44)	11 (46)	0.92
Pulmonary hypertension, *n (%)*	13 (25)	9 (33)	5 (21)	0.32
Digital ulcer, *n (%)*	34 (67)	19 (70)	15 (63)	0.55
Telangiectasias, *n (%)*	22 (43)	14 (52)	8 (33)	0.18
Autoantibodies				
Anti-RNA pol III, *n (%)*	13 (25)	10 (37)	3 (13)	0.04
Anti-Scl70, *n (%)*	15 (29)	9 (33)	6 (25)	0.51
Anti-centromere, *n (%)*	12 (24)	0 (0)	12 (50)	< 0.001
Anti-PM-Scl, *n (%)*	5 (10)	1 (4)	4 (17)	0.12
ANA, *n (%)*	51 (100)	27 (100)	24 (100)	-
Treatments before SRC				
ACEi before SRC, *n (%)*	9 (18)	6 (22)	3 (13)	0.27
CS before SRC, *n (%)*	15 (29)	11 (41)	4 (17)	0.06
Non-CS immunosuppressant, *n (%)*	26 (51)	16 (51)	10 (42)	0.21

ACEi, angiotensin-converting enzyme inhibitors; ANA, antinuclear antibodies; Anti-RNA pol III, anti-RNA polymerase III; BP, blood pressure; CS, corticosteroids; DLCO, diffusing capacity of carbon monoxide; IQR, interquartile range; mRSS, modified Rodnan score; non-CS immunosuppressants, include methotrexate, cyclophosphamide, and rituximab; PM-Scl, polymyositis-scleroderma; SRC, scleroderma renal crisis; SSc, systemic sclerosis.

Results are presented as median [IQR] unless otherwise specified.

**Table 2:** PlGF, sFlt-1, and renin dosage in patients with microangiopathy compared with controls

Characteristics	Donors (*n* = 61)	All SSc (*n* = 51)	SSc SRC+ (*n* = 27)	SSc SRC-(*n* = 24)	HUS (*n* = 27)	MHT (*n* = 22)	*P*-value
PlGF median [IQR] (pg/ml)	14.7 [11.8–17.9]	22 [15.8–42.9]	42.1 [21.4–51.8]	18.5 [14.7–21.5]	22.8 [19.5–29.6]	25.5 [17.2–39.3]	< 0.0001
*n* (%) > Mean + 2SD donors = 26.51	3 (4.92)	18 (35.3)	17 (63.0)	1 (4.17)	10 (37.0)	10 (45.5)	< 0.0001
sFlt-1 median [IQR] (pg/ml)	84 [77.4–98.2]	86 [75.8–113]	102 [84.3–130]	84 [74.2–86.8]	116 [101–206]	117 [93.3–140]	< 0.0001
n (%) > Mean + 2SD donors = 130.82	4 (4.56)	7 (13.7)	6 (22.2)	1 (4.17)	9 (33.3)	9 (40.9)	< 0.0001
Ratio sFlt-1/PlGF median [IQR]	6.09 [4.82–7.89]	4.32 [2.38–5.8]	3.68 [2.14–5.57]	4.80 [3.56–5.86]	5.29 [3.82–7.12]	4.36 [3.15–6.14]	< 0.0001
*n* (%) > Mean + 2SD donors = 12.86	3 (4.92)	1 (1.96)	1 (3.70)	0 (0)	3 (11.1)	1 (4.55)	0.42
Renin median [IQR] (pg/ml)	-	137 [47–380]	392 [218–2020]	47 [19–99]	-	-	< 0.001
Renal dysfunction							
Creatininemia median [IQR] (μmol/l)	-	99 [65–267]	254 [115–391]	66 [59–85]	398 [166–752]	400 [213–711]	< 0.0001
Proteinuria median [IQR] (g/g)	-	0.1 [0.1–0.3]	0.5 [0.2–1.1]	0.1 [0.1–0.1]	2.3 [1.2–8.1]	0.9 [0.2–2.5]	< 0.0001
Microangiopathic hemolytic anemia							
Hemoglobin median [IQR] (g/dl)	-	11.7 [9.7–13.4]	9.8 [8.7–12.2]	13.0 [11.5–14.3]	8.0 [6.6–9.6]	9.8 [6.9–11.9]	< 0.0001
Platelets median [IQR] (G/l)	-	250 [106–296]	129 [85–254]	286 [243–327]	61 [25–100]	102 [78–141]	< 0.0001
Schistocytes median [IQR] (%)	-	0 [0–1]	1 [0–1]	0 [0–0]	1 [0–2]	0 [0–1]	< 0.0001
LDH median [IQR] (UI/l)	-	241 [200–427]	435 [300–886]	205 [185–232]	1265 [388–3366]	579 [412–995]	< 0.0001
Haptoglobin median [IQR] (g/l)	-	0.9 [0.2–1.4]	0.1 [0–0.6]	1.3 [0.9–1.7]	0 [0–0]	0 [0–0.2]	< 0.0001
Albumin median [IQR] (g/l)	-	42 [40–44]	41 [30–44]	42 [40–45]	27 [28–30]		0.001
NT Pro BNP median [IQR] (ng/l)	-	84 [14–962]	4666 [984–6855]	62 [0–126]	-	-	0.001
Troponin median [IQR] (ng/l)	-	0 [0–41]	107 [7–321]	0 [0–7]	16 [6–431]	1 [0–3]	< 0.001
CRP median [IQR] (mg/l)	-	3 [0–12]	11 [0–65]	2 [0–6]	52 [5–100]	21 [0–21]	0.03
	-						
ACE inhibitor, *n* (%)	-	30 (59)	27 (100)	3 (13)	3 (11)	5 (23)	< 0.0001
Dialysis, *n* (%)	-	12 (24)	11 (41)	1 (4)	7 (26)	3 (4)	0.03

ACE inhibitor, angiotensin converting enzyme inhibitor; HUS, hemolytic uremic syndrome; LDH, lactic acid dehydrogenase; MHT, malignant hypertension; PlGF, placental growth factor; sFlt-1, soluble fms-like tyrosine kinase 1; SSc, systemic sclerosis; SSc-SRC−, SSc patient with no history of scleroderma renal crisis; SSc-SRC+, SSc patient with scleroderma renal crisis.

Representation of mean dosages of biomarkers (PlGF, sFlt-1 and Renin) among patients with SSc with and without history of SRC, compared with donors, HUS and MHT patients. Means are represented with 2 SDs compared with donors’ group. Medians are represented with the interquartile range [IQR].

*P*-values were assessed using nonparametric Kruskal Wallis test for continuous values, and Chi-2 statistic was used for discrete values (presented as *n* [%]).

### Measurement of Circulating sFlt-1 and PlGF in SRC and Controls

**Figure 1:**
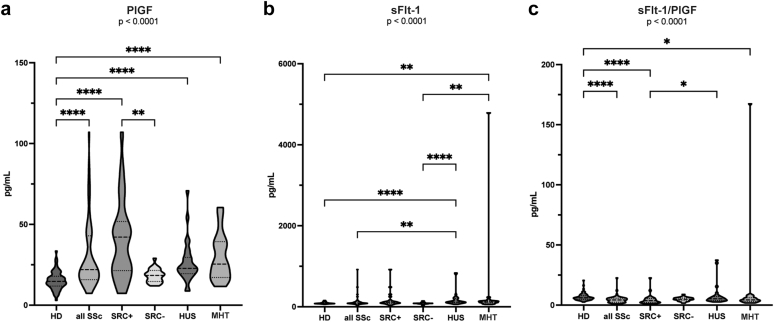
(a) PlGF, (b) sFlt-1 concentrations and (c) sFlt-1/PlGF ratio in patients with SRC versus controls. PlGF levels were significantly higher in patients with SSc-SRC+ than in controls. sFlt-1 and sFlt-1/PlGF ratio were not significantly different between SSc-SRC+ and SSc-SRC−. D, donors; HUS, hemolytic uremic syndrome; MHT, malignant hypertension; PlGF, placental growth factor; sFlt-1, soluble fms-like tyrosine kinase1; SRC, scleroderma renal crisis; SSc, systemic sclerosis.

The median delay between SRC occurrence and serum sample collection was 0.42 (0.2–1.5) months. Circulating sFlt-1, PlGF, and ratio values are reported in Table 2 and Figure 1. PlGF was significantly higher in the SSc-SRC+ group with a median of 42.1 (21.4–51.8) pg/ml in comparison to the SSc-SRC− group, which presented a median level of 18.5 (14.5–21.5) pg/ml (*P* < 0.0001). Moreover, PlGF levels were higher among patients with SSc with a median (IQR) of 22 (16.8–42.9) pg/ml, compared with donors who had a median level of 14.7 (11.8–17.9) pg/ml (*P* < 0.001). Similar patterns were observed when comparing patients with SSc-SRC+ to those with HUS (22.8 [19.5–29.6] pg/ml) and those with MHT (25.5 [17.2–39.3] pg/ml), in which PlGF levels were significantly lower (*P* < 0.001). When comparing PlGF levels exclusively in patients with dcSSc (dcSSc-SRC+: *n* = 21 and dcSSc-SRC−: *n* = 6), we found that PlGF levels remained significantly higher in the dcSSc-SRC+ group, with a median (IQR) of 43 (25–55) pg/ml compared with a median of 19 (18–19) pg/ml in the dcSSc-SRC− group (*P* = 0.005). Similarly, when comparing PlGF levels among anti-centromere antibody-negative patients, and among hypertensive patients, PlGF levels remained increased in the SSc-SRC+ group (*P* = 0.005 and *P* = 0.045, respectively). In addition, we found no significant difference when comparing PlGF levels between patients with SSc with positive or negative anti-RNA polymerase III antibody, status (*P* = 0.4), or between those with or without steroid exposure (*P* = 0.7). Furthermore, clinical features such as PH (*P* = 0.5) and digital ulcers (*P* = 0.6) did not influence PlGF levels. Patients were classified as high PlGF (*n* = 18, 35%) when they had a PlGF measurement above 26.5 pg/ml at baseline (defined using the mean + 2 SDs). Patients with high PlGF had a higher prevalence of diffuse forms of SSc (*n* = 15, 83%) compared with the patients with lower PlGF (*n* = 12, 36%) (*P* = 0.005). Among 18 patients with high PlGF, 3 (2%) presented with limited cutaneous SSc. The proportion of patients with telangiectasia (*n* = 11, 65%) was also significantly higher among patients with high PlGF levels (*P* = 0.034). sFlt-1 levels were not statistically different between SSc-SRC+ and SSc-SRC− groups, with a median concentration of 102 (84.3–130) pg/ml and 84 (74.2–86.8) pg/ml, respectively (*P* > 0.05). We did not find a significant difference in sFlt-1/PlGF ratio between SSc-SRC+ and SSc-SRC− groups (*P* > 0.05), and with respect to patients with MHT and those with HUS (*P* > 0.05).

### Diagnostic Performance of Serial Biomarker Levels

**Figure 2:**
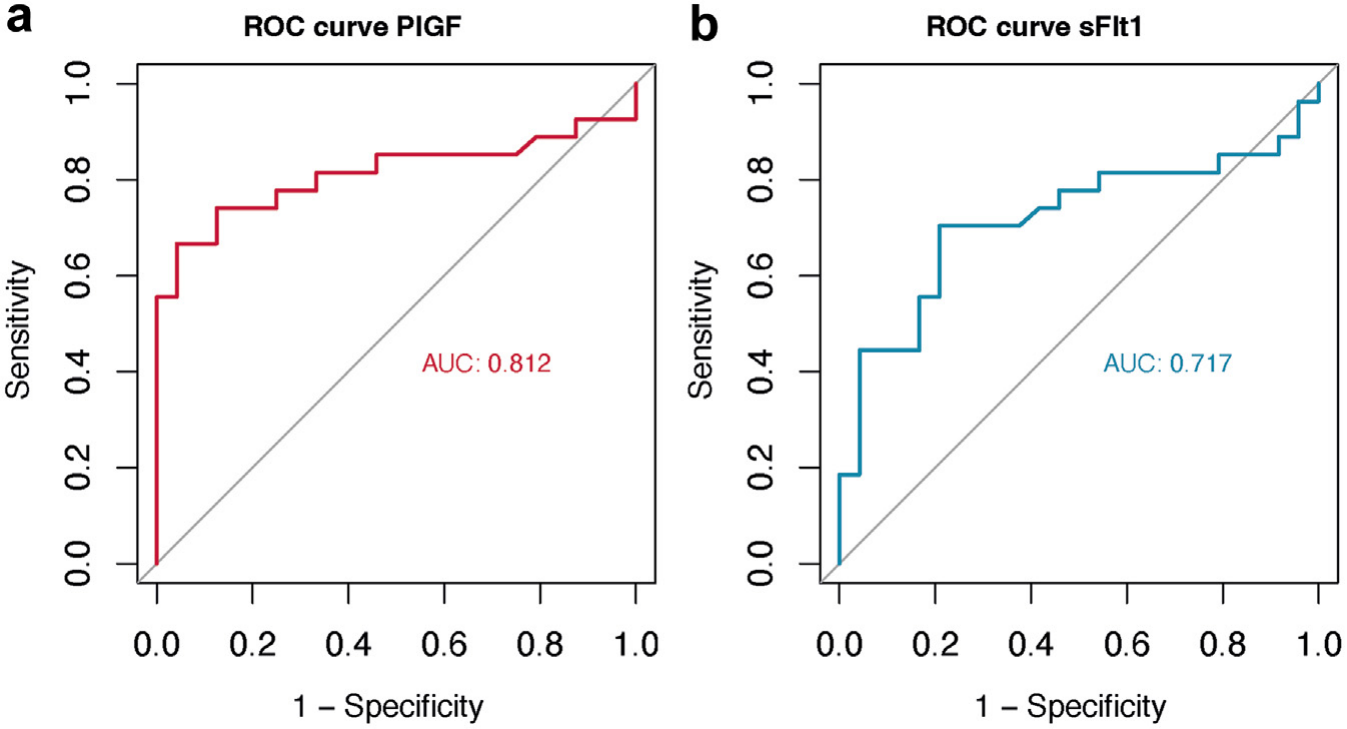
Receiver operating characteristic (ROC) curves of sFlt-1 and PlGF, in SRC versus non-SRC patients.(a) ROC curve for PlGF (AUC: 0.81 [confidence interval, CI: 0.68–0.94]). (b) ROC curve for sFlt-1 (AUC: 0.72, [CI: 0.57–0.87]). A PlGF threshold of 24.5 pg/ml was associated with a specificity of 95% and a sensitivity of 67%. No threshold was established for sFlt-1 because of insufficient sensitivity and specificity. AUC, area under the curve; PlGF, placental growth factor; ROC,receiver operating characteristics; sFlt-1, soluble fms-like tyrosine kinase 1; SRC, scleroderma renal crisis.

We evaluated the diagnostic performance of PlGF and sFlt-1 for SRC identification among patients with SSc using receiver-operating-characteristic curves. Using a multivariate logistic regression, SRC remained independently associated with steroid exposure (*P* = 0.017) and PlGF (*P* = 0.034) (Supplementary Table S1). The diagnostic accuracy of PlGF measurement for SRC diagnosis, expressed as an AUC, was 0.81 (95% confidence interval: 0.68–0.94). The AUC of sFlt-1 for SRC diagnosis, was 0.72 (0.56–0.86) (Figure 2b). A PlGF threshold of 24.5 pg/ml was associated with a specificity of 95% and a sensitivity of 67%. Regarding the diagnostic performance of sFlt-1/PlGF ratio, we observed an AUC of 0.67 (0.51–0.82) (Supplementary Figure S1). Considering the lower AUC value and insufficient sensitivity and specificity, we did not determine a threshold for both sFlt-1 and sFlt-1/PlGF ratio.

### Association Between PlGF and Renal Prognosis

**Figure 3:**
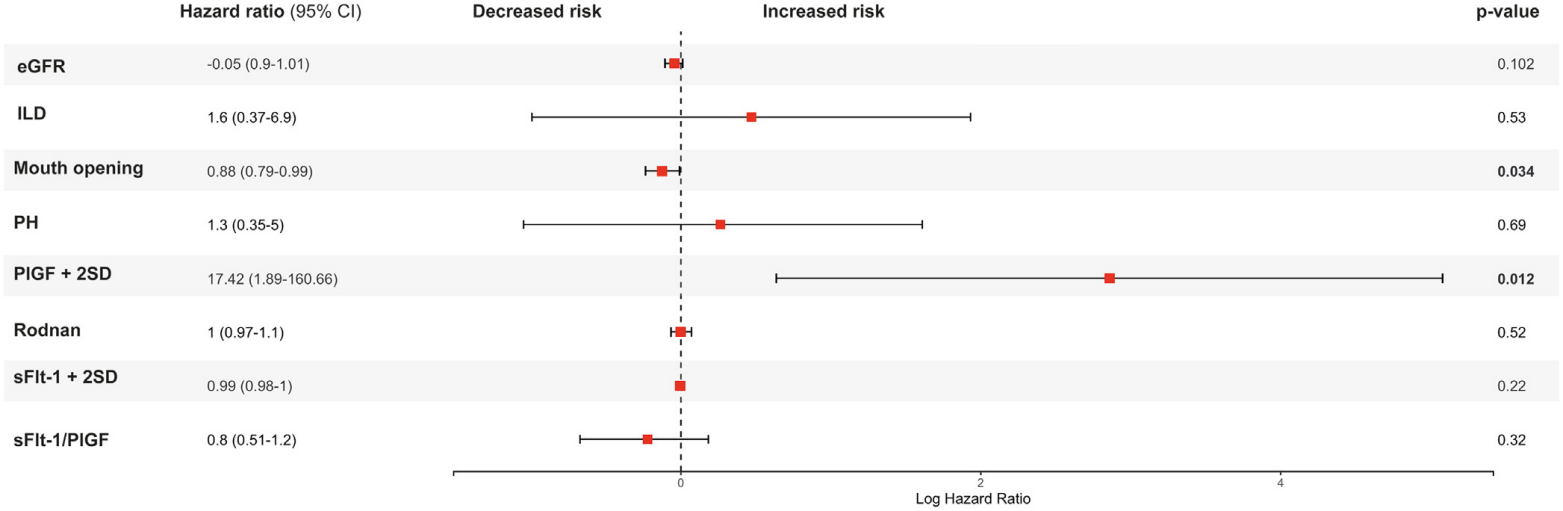
Forest plot representation of variables associated with an increased risk of long-term dialysis. A natural logarithmic scale was used on the x-axis. PlGF was significantly associated to an increased risk of long-term dialysis. PlGF + 2 SDs and sFlt1 + 2 SDs were used. Estimates include hazard ratio (95% confidence intervals). eGFR, estimated glomerular filtration rate; PlGF, placental growth factor; SD, standard deviation.

Higher PlGF levels were associated with a higher risk of chronic kidney disease in a Cox model analysis, with a hazard ratio of 17.41 (1.88–160; *P* = 0.012). This performance was superior to that of the estimated glomerular filtration rate, which showed a hazard ratio of 0.95 (0.9–1.10; *P* = 0.102) (Figure 3). Creatinine measurements were missing for 3 patients with SSc. After a median (IQR) follow-up time of 6 (2–11) years, a total of 11 (41%) SSc-SRC+ patients required dialysis, 3 (27%) acutely (discontinuing it within 3 months), whereas 8 (73%) required long-term dialysis (> 3 months). Long-term dialysis status was missing for 1 patient. A total of 4 patients received kidney transplantation. In the overall SSc population, PlGF levels were not statistically different in transplanted patients, with a median of 23.4 (21.9–38.1) pg/ml, compared with a median PlGF level of 20 (15.8–43.4) pg/ml among the nontransplanted patients with SSc (*P* = 0.48). PlGF levels did not significantly vary according to anti-RNA polymerase III antibody status among patients with SSc-SRC+ (PlGF median [IQR] levels at 43 [22–50] pg/ml among antibody negative patients, and 29 [21–44] pg/ml among antibody-positive patients). Contrary to PlGF, sFlt-1 (with a hazard ratio of 1 [1–1]) and sFlt-1/PlGF ratio (with a hazard ratio of 0.8 [0.5–1.2]) were not associated to an increased risk of long-term dialysis among patients with SSc in our Cox model analysis (Figure 3). In our study cohort, death occurred in 8 patients (16%) with SSc, among them 5 patients with SSc-SRC+ and 4 with SSc-SRC−.

## Discussion

This study provides evidence supporting an association between PlGF elevation and SRC. We show that levels of PlGF are increased in patients with SRC and in patients with SSc who are at risk of long-term dialysis. To the best of our knowledge, this study is the first to report a link between PlGF increase and poor renal prognosis among patients with SRC.

Predictive factors for SRC occurrence have been previously sought in patients with SSc. Clinical and biological factors, such as glucocorticoid treatment > 7.5 mg daily with a dose-dependent effect, anti-RNA polymerase III antibodies, tendon friction rubs, and synovitis^2^ have been identified. Conversely, preexisting renal function abnormalities such as isolated hypertension and proteinuria alone were not identified as SRC biomarkers.^18^ Other SRC biomarkers, including soluble adhesion molecules sE-selectin, soluble vascular cell adhesion molecule-1, and soluble intercellular adhesion molecule-1,^19^ soluble CD147,^20^ endostatin,^21^ orendothelin-1,^22^ have also been identified but are expensive and time consuming to measure and, thus, not routinely used in clinical practice. In addition, none of these biomarkers were reported to predict the risk of end-stage kidney disease and dialysis. Overall, novel biomarkers for SRC are still needed.

Owing to physiological and phenotypical similarities between SRC and preeclampsia, we proceeded to compare PlGF, sFlt-1, and their ratio as diagnostic and prognostic biomarkers of SRC. sFlt-1 and PlGF are angiogenesis biomarkers routinely used in clinical practice to diagnose preeclampsia based on an early decrease in PlGF and a delayed increase in sFlt-1.^23^^,^^24^ Associations between PlGF and vasculopathies, including diabetic retinopathy,^25^ ischemic cardiomyopathy, limb ischemia, and stroke,^26^ have previously been reported. Preeclampsia is a potentially life-threatening disease affecting women in the later stages of pregnancy. SRC and preeclampsia share similarities in their clinical phenotypes, both presenting with acute kidney injury, microangiopathic hemolytic anemia,^27^ and elevated blood pressure. The pathophysiology of preeclampsia involves placental dysfunction leading to endothelial injury, reduced vasodilation, increased vasoconstriction, and inflammation,^10^ similarly to SRC, where endothelial cell injury and activation as well as glomerular ischemia are hallmarks of the disease. The sFlt1/PlGF ratio has been validated as a diagnosis tool in women with suspected preeclampsia, with a negative predictive value of 99.3% for a threshold of 38.^23^ Based on these phenotypical similarities, we aimed to study the use of angiogenesis biomarkers PlGF and sFlt-1 in SRC

Contrary to our initial assumption, PlGF and sFlt-1 levels in SRC differed from those observed in preeclampsia.^24^^,^^28^ These differences may be explained by the abnormal production of sFlt-1 by placental cytotrophoblasts in preeclampsia. In preeclampsia, increased production of sFlt-1 is initially triggered by pregnancy, but becomes further dysregulated, leading to excessive sFlt-1 levels and decreased PlGF compared with healthy pregnancy.^29^ In contrast, in SRC, sFlt-1 levels were similar to those seen in healthy controls, suggesting a more regulated or normal production. This lack of increased sFlt-1 production in SRC may explain why free PlGF is not suppressed in this condition. It is also important to note that PlGF levels remain higher in both SRC and preeclampsia compared with nonpregnant healthy controls.

Angiogenesis and inflammation are intertwined and interdependent processes, a fact that can contribute to the higher levels of PlGF observed in patients with SSc compared with donors. Evidence suggests a crosstalk between T cells and endothelial cells, with PlGF acting as “angio-lymphokine”, inducing proinflammatory Th17 differentiation and dampening of regulatory T cells (TREG) activation.^30^ There is also documentation indicating an imbalance of Th17-to-regulatory T cells ratio playing a role in the pathogenesis of SSc through cytokine secretion.^30^, ^31^, ^32^

sFlt-1 alone and sFlt-1/PlGF ratio displayed poor diagnostic performance in our cohort, which does not support their use to diagnose or assess prognosis following SRC. Notably, sFlt-1 levels were significantly lower in our patient cohorts compared with the levels observed in women with ongoing pregnancies, with or without preeclampsia. Mean (± SD) sFlt-1 levels as high as 4079 (± 1652) pg/ml have been previously reported in healthy pregnant women, and over 7000 (± 509) pg/ml in women affected with preeclampsia.^33^

In our cohort, we found an association between higher PlGF levels and the occurrence of SRC. PlGF is a proangiogenic molecule with structural and physiological similarities with vascular endothelial growth factor. PlGF molecule was first cloned from a library of complementary DNA derived from human placenta.^34^ PlGF is also expressed at lower levels in many other tissues, including the heart, lung, thyroid, liver, and muscles.^35^The molecule presents as a glycolyzed heterodimer with 4 isoforms obtained through alternative splicing and selectively binds to Flt-1. Variations in PlGF levels have been described in various pathological conditions, highlighting its role as a multitasking cytokine with pleiotropic effects, beyond vasculopathies alone; its role has been extensively described in malignancy studies, as well as in atherosclerosis^36^ and rheumatoid arthritis.^37^ Interestingly, we found a higher incidence of telangiectasia, a visible manifestation of vasculopathy, among patients with SSc with high PlGF levels. Contrary to a previous case study on PlGF in SSc-associated PH,^38^ we did not find a higher incidence of PH among patients with high PlGF, possibly because of a lack of power with only 13 patients (25%) presenting with PH in our cohort. Overall, these previous studies, along with ours, suggest that elevated levels of PlGF may be linked with SSc vasculopathy.

Interestingly, we found that the increased PlGF levels were associated with an increased risk of long-term dialysis in patients presenting with SRC. In the literature, kidney disease is known to be linked with augmented PlGF levels, which are significantly associated with mortality and cardiovascular events, likely through increased vascular inflammation.^39^ This association was also found in our study, in which patients with impaired renal function in the HUS, MHT, or SRC groups had higher PlGF levels compared with the non-SRC group or donors. Though the mechanisms of PlGF elevation in the context of chronic kidney disease are unclear, one hypothesis implies an upregulation of PlGF through toxin accumulation or renin-angiotensin-aldosterone stimulation. Alternatively, PlGF elevation could be attributed to a decrease in sFlt-1 levels, which typically antagonizes PlGF in endothelial cell walls.^40^ Molecular weight of PlGF is low (34 kDa) and hence its clearance may theoretically be affected by the estimated glomerular filtration rate. We may not rule out that the rapid deterioration of renal function which defines SRC contributes to explaining why PlGF increases in this context. The correlation between PlGF and serum creatinine was only borderline significant, and the magnitude of PlGF increase observed in patients with SRC far exceeds what is expected in patients with an estimated glomerular filtration rate < 30 ml/min per 1.73 m^2^ (Supplementary Figure S2).^40^ The much clearer correlation between renin and PlGF suggests that renal ischemia induced by the endothelial lesions of scleroderma reflects what triggers PlGF synthesis, not clearance (Supplementary Figure S3). The higher levels of PlGF in patients with SRC compared with patients with HUS and patients with MHT are in favor of an inflammation-fueled process, possibly leading to endothelial injury triggering or sustaining SRC.

The strengths of our study include the comparison of patients with SSc, donors, and with other causes of thrombotic microangiopathy to assess the specificity of marker evaluation. However, there are some limitations to our study stemming from the observational and retrospective design, making it challenging to establish a cause-and-effect relationship between PlGF increase and SRC. Foremost, we acknowledge the possibility of the remaining confounding bias because of the limited comparability between patients with SSc-SRC+ and those with SSc-SRC−regarding risk factors for SRC occurrence. Although the multivariate regression analysis identified a significant association between PlGF and the risk of SRC, we recognize that the low number of events may have introduced imprecision in our model. In addition, patients with SSc were recruited from a national tertiary referral center for autoimmune diseases, which could potentially limit the external validity of our result and would also require external validation. Furthermore, Butler criteria were used to classify patients as SSc-SRC+, though international efforts are ongoing to this day to establish consensual classification criteria for SRC. Even if we compared PlGF levels in patients with similar renal and clinical conditions (HUS and MHT), future studies should aim to compare PlGF in SSc-SRC+ patients who do not fulfill Butler *et al* classification criteria,^13^ particularly in case of normotensive presentation. Although our results show a possible use of PlGF as a diagnostic and prognostic biomarker for SRC, PlGF measurements were not available before the onset of SRC. Therefore, future research should include samples collected before SRC onset to accurately evaluate the biomarker’s potential for predicting SRC and a longitudinal collection of sera samples to establish the predictive role of the angiogenesis molecule.

## Conclusion

Our study reports that high PlGF levels are associated with SRC and the risk of kidney failure. These findings suggest that PlGF could serve as a valuable biomarker for SRC renal prognosis with no additional interest in measuring sFlt-1 and in calculating the sFlt-1/PlGF ratio. However, prospective long-term studies including sera assessment before SRC are needed to validate the use of PlGF as a reliable diagnostic biomarker for SRC in clinical practice.

## Disclosure

All the authors declared no conflicting interests.

## Supplementary Material

Supplementary File (PDF) **Figure S1.** Receiver operating characteristic (ROC) curve of sFlt-1/PlGF ratio. **Figure S2.** Correlation between PlGF and creatinine levels. **Figure S3.** Correlation between PlGF and renin. **Table S1.** Multivariate analysis of factors independently associated with scleroderma renal crisis in patients with SSc.

## Footnotes

Supplementary File (PDF)
**Figure S1.** Receiver operating characteristic (ROC) curve of sFlt-1/PlGF ratio. **Figure S2.** Correlation between PlGF and creatinine levels. **Figure S3.** Correlation between PlGF and renin. **Table S1.** Multivariate analysis of factors independently associated with scleroderma renal crisis in patients with SSc.
