# Supplementary material for: Pilot Study of Diagnostic Performances of Vascular Biomarkers Soluble fms-Like Tyrosine Kinase and Placental Growth Factor in Scleroderma Renal Crisis
Source: Kidney Int Rep. 2024 Dec 31;10(3):866–76. doi: 10.1016/j.ekir.2024.12.025 (PMC11993226; doi:10.1016/j.ekir.2024.12.025)
Supplement: Supplementary File (PDF) — Figure S1. Receiver operating characteristic (ROC) curve of sFlt-1/PlGF ratio. Figure S2. Correlation between PlGF and creatinine levels. Figure S3. Correlation between PlGF and renin. Table S1. Multivariate analysis of factors independently associated with scleroderma renal crisis in patients with SSc. [file mmc1.pdf]

Supplementary Material

Supplementary Figures

Supplementary Table S1: Multivariate analysis of factors independently associated with scleroderma renal crisis in SSc patients

Supplementary Figure S1: Receiver Operating Characteristic (ROC) curve of sFlt-1/PlGF Ratio

Supplementary Figure S2. Correlation between PlGF and Creatinine Levels

Supplementary Figure S3. Correlation between PlGF and Renin

(PDF)

Supplementary information is available at KI Report's website

Supplementary Table S1: Multivariate analysis of factors independently associated with scleroderma renal crisis in SSc patients

Parameter	Odds Ratio	95% confidence interval	p-value
PlGF	1.08	[1.01-1.22]	**0.034**
dcSSc	1.24	[0.16-7.36]	0.898
Anti-RNA pol III	3.27	[0.44-25.24]	0.193
High blood pressure	3.35	[0.35-36.31]	0.281
Telangiectasias	1.34	[0.21-8.33]	0.745
Steroid exposure	28.46	[1.65-5821]	**0.017**

Multivariate regression analysis performed to determine factors independently associated with scleroderma renal crisis in SSc patients. *Anti-RNA pol III: Anti RNA polymerase III; dcSSc: diffuse cutaneous systemic sclerosis; PlGF: placental growth factor.* (PDF)

Supplementary Figure S1: **Receiver Operating Characteristic (ROC) curve of sFlt-1/PlGF Ratio.**

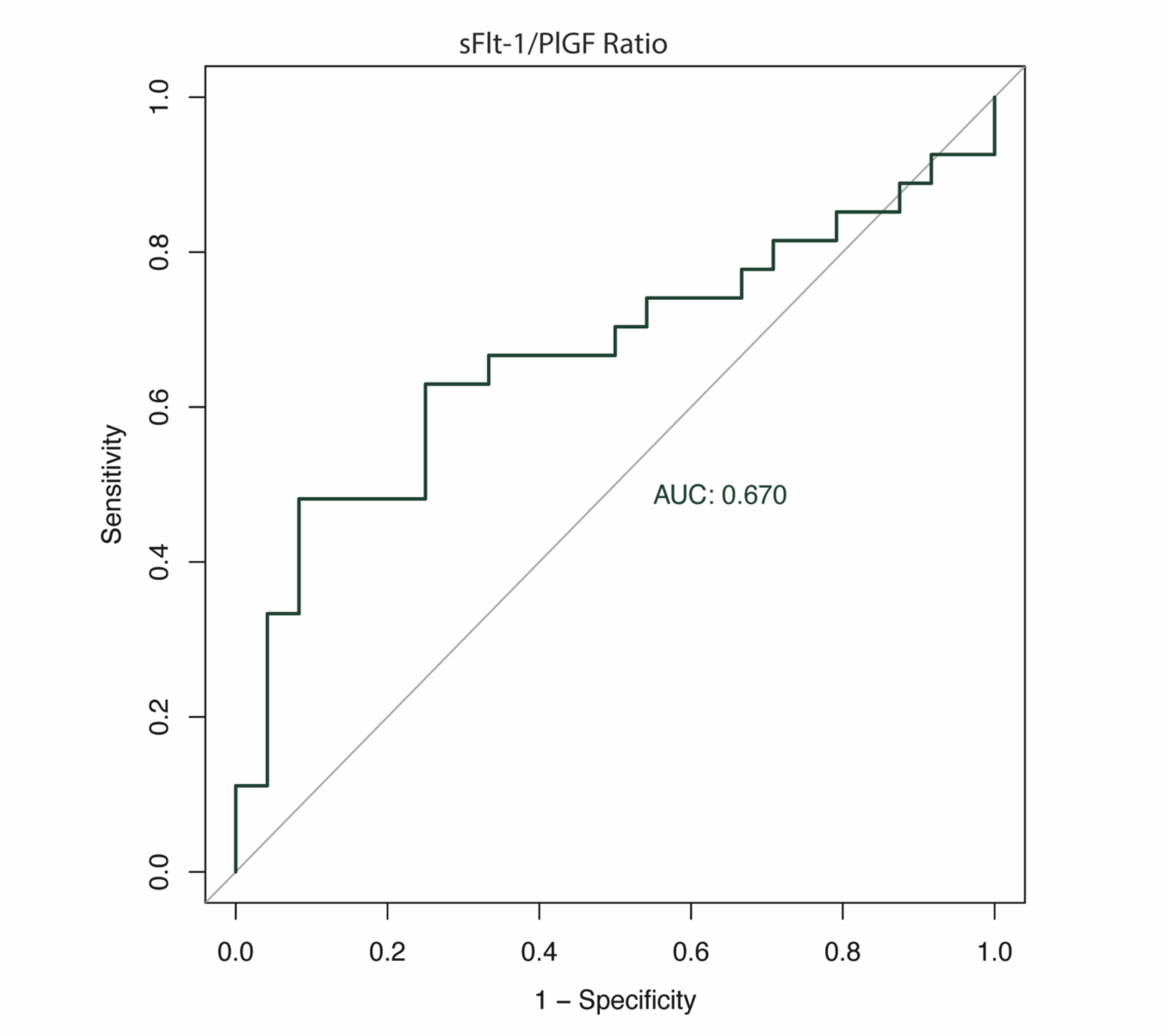

ROC curve of sFlt-1/PlGF ratio comparing performances between SSc SRC+ and SSc SRC- patients. (AUC = 0.67, CI [0.51 – 0.82]). *No threshold was established for sFlt-1/PlGF ratio due to insufficient sensitivity and specificity; AUC: area under curve; PlGF: placental growth factor; ROC: Receiver operating characteristic; sFlt-1: soluble fms-like tyrosine kinase 1.* (TIFF).

Supplementary Figure S2. Correlation between PlGF and Creatinine Levels.

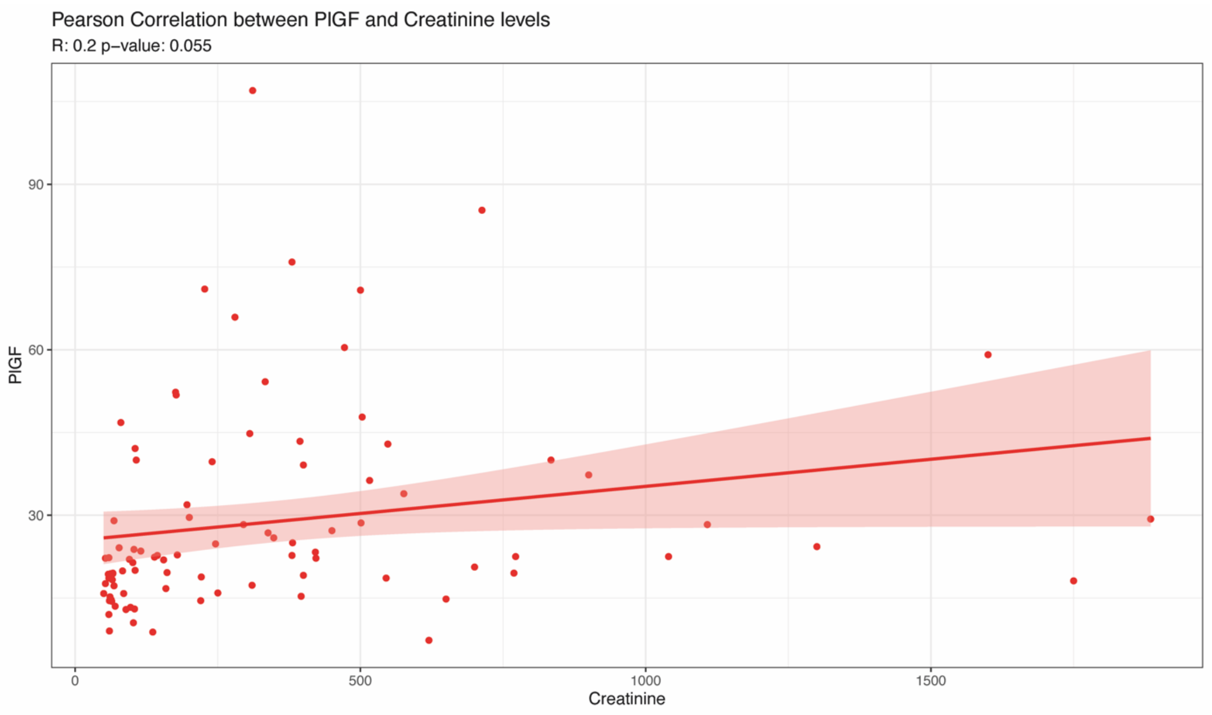

Pearson correlation representation of correlation between PlGF and serum creatinine, showing a borderline significant association. *PlGF: placental growth factor.* (TIFF).

Supplementary Figure S3. Correlation between PlGF and Renin.

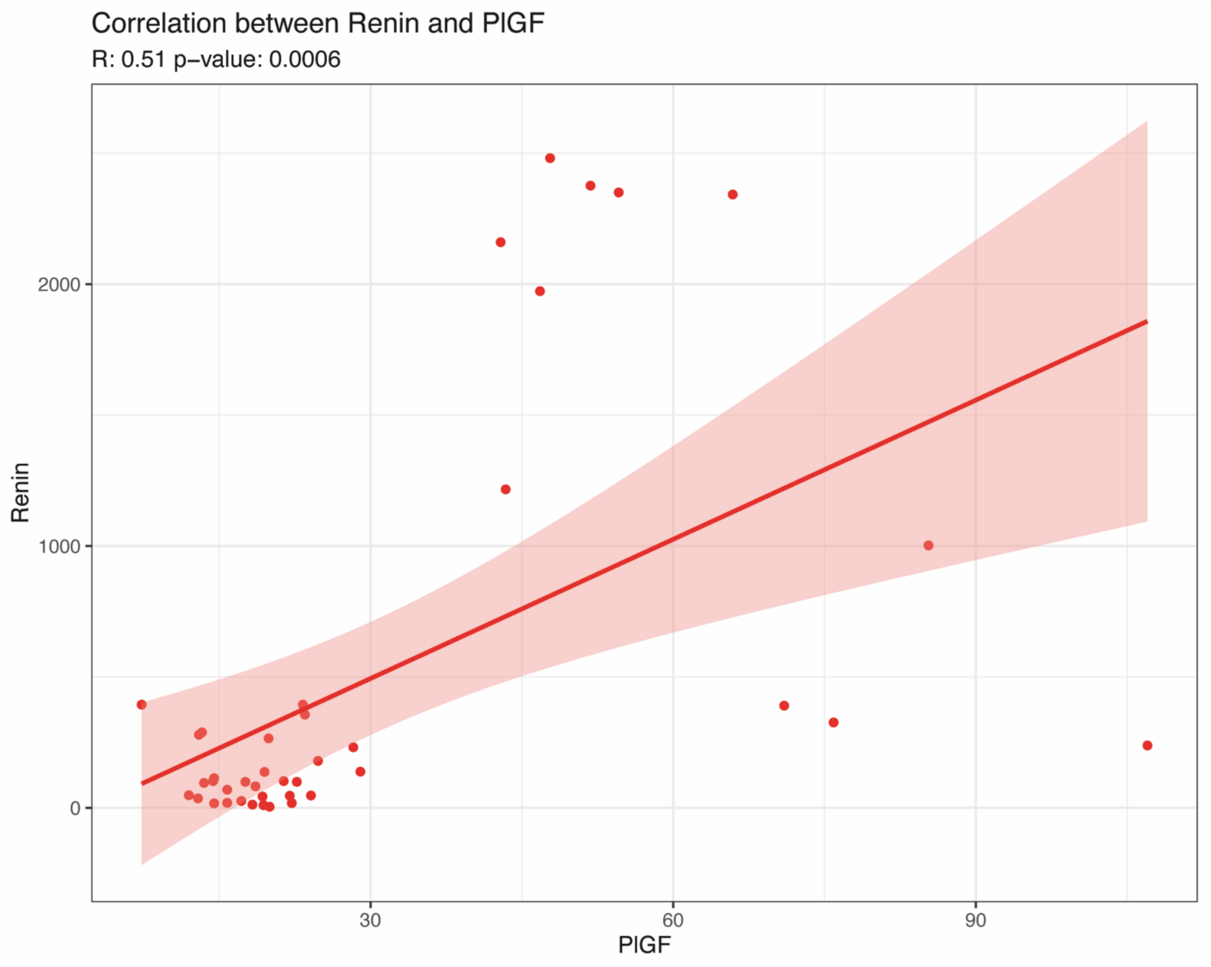

Pearson correlation representation of correlation between PlGF and Renin, showing a significant association. *PlGF: placental growth factor.* (TIFF).
